# Regulation of Vascular Function on Posttranscriptional Level

**DOI:** 10.1155/2013/948765

**Published:** 2013-10-31

**Authors:** Andreas Eisenreich

**Affiliations:** Charit-Universitätsmedizin Berlin, CCM, Institut of Clinical Pharmacology and Toxicology Berlin, Charite Platz 1, 10117 Berlin, Germany

## Abstract

Posttranscriptional control of gene expression is crucial for regulating plurality of proteins and functional plasticity of the proteome under (patho)physiologic conditions. Alternative splicing as well as micro (mi)RNA-mediated mechanisms play an important role for the regulation of protein expression on posttranscriptional level. Both alternative splicing and miRNAs were shown to influence cardiovascular functions, such as endothelial thrombogenicity and the vascular tone, by regulating the expression of several vascular proteins and their isoforms, such as Tissue Factor (TF) or the endothelial nitric oxide synthase (eNOS). This review will summarize and discuss the latest findings on the (patho)physiologic role of alternative splicing processes as well as of miRNAs on modulation of vascular functions, such as coagulation, thrombosis, and regulation of the vascular tone.

## 1. Introduction

The expression of proteins and their isoforms is of immense importance for the induction as well as the control of (patho)physiologic functions in the vasculature, such as maintenance of the vessel wall homeostasis, blood coagulation, thrombosis, modulation of the vascular tone, and angiogenesis [1–8]. The differential protein and isoform expression is highly regulated on transcriptional as well as on posttranscriptional level. The regulatory factors and mechanisms of gene expression on transcriptional level were reviewed in detail elsewhere [9–11]. Therefore, this review will focus on the posttranscriptional expression regulation and the influence of these processes on vascular function. 

The modulation of gene expression on posttranscriptional level is essential for increasing and for regulating the diversity of proteins and their biologic functions under (patho)physiologic conditions [12, 13]. Alternative splicing and micro (mi)RNA-mediated processes are the most important mechanisms for the control of protein expression on posttranscriptional level [14, 15]. Moreover, both mechanisms were demonstrated to control vascular functions (see Tables 1 and 2), such as endothelial thrombogenicity and regulation of vascular tone, by modulating the expression of vascular proteins, such as Tissue Factor (TF) and endothelial nitric oxide synthase (eNOS) [4, 8, 16–19]. The following parts of the paper will briefly summarize the latest findings regarding the influence of alternative splicing and miRNAs on the expression and function of vascular factors, such as TF and eNOS. 

## 2. The Impact of Posttranscriptional Expression Regulation on Vascular Functions

There is a great discrepancy between the number of estimated protein-coding genes in the human genome (approximately 24,000) and the number of generated proteins (about 100,000) [20]. Posttranscriptional expression modulation via alternative splicing as well as miRNAs-mediated control is a major contributor to this immense increase in protein diversity [20, 21]. 

In general, alternative splicing and miRNAs regulate protein expression on posttranscriptional level [12–15]. However, some processes involved in alternative splicing control were recently indicated to occur rather cotranscriptionally, such as assembly of the spliceosome-mediated excision of introns from growing primary RNA chains tethered to their coding DNA as identified by electron *microscope* studies or the recruitment of the splicing factor serine/arginine-rich splicing factor (SRSF)3 to the primary transcript of fibronectin by RNA polymerase II, which consequently leads to reduced inclusion of alternative exons into the mature fibronectin mRNA [22].

It was suggested that about 70% of all human genes are alternatively spliced [12]. This mechanism of post-translational expression control leads to the generation of several mature mRNA splice variants and protein isoforms which can differ in their intracellular localization, binding affinity, and activity from other isoforms [1, 8, 12]. The resulting variability of protein isoforms—in turn—increases the cellular repertoire and possibility of fine tuning of different biologic functions in general and especially in the vasculature (see Table 1) [4, 23]. 

miRNA-mediated expression regulation is also an important control mechanism which modulates the functional properties of cells and tissues [21, 24]. It was assumed that miRNAs control approximately 30% of all human protein-coding genes [25]. In contrast to alternative splicing which modulates the isoform expression at sites of mRNA synthesis and processing within the nucleus, miRNAs regulate the expression of mature mRNAs in the cytoplasm [12, 21, 25]. Moreover, miRNAs most often mediate repression of the expression of corresponding targets (see Table 2) [13]. 

The following part of the paper will illustrate the modulatory role of alternative splicing and miRNAs in basic (patho)physiologic-relevant vascular processes, such as blood coagulation, thrombosis, and regulation of vascular tone.

## 3. Differential Impact of Alternatively Spliced Isoforms in the Vasculature

### 3.1. Thrombosis and Blood Coagulation

The blood coagulation cascade is of immense importance for a variety of (patho)physiologic-relevant vascular processes, such as vessel wall homeostasis, wound healing, and thrombosis [3, 18]. In consequence, this leads to the basic necessity that this biologic process is highly regulated. Alternative splicing as well as miRNA-mediated regulation of protein expression were demonstrated to be important modulators of blood coagulation and thrombosis (see Tables 1 and 2) [3, 16, 18]. 

#### 3.1.1. TF

TF is the primary initiator of blood coagulation [11, 26]. Due to alternative splicing, three mRNA splice variants were expressed in the vasculature [1]. “Full-length” (fl)TF is the longest mature transcript [2]. Translation of this mRNA species results in the generation of membrane-bound flTF protein, which is highly prothrombogenic [2, 18, 27]. The second mRNA isoform is named “alternatively spliced” (as)TF. Translation of this shorter mRNA variant leads to the formation of a soluble protein isoform [2]. The procoagulant activity of asTF is very low [2, 18]. FlTF, rather than asTF, was shown to be the main source of procoagulant TF activity [2, 27], whereas, asTF was recently linked more closely to proangiogenic processes and cell proliferation [6, 28, 29]. A third mRNA splice variant was named TF-A. This isoform was found to be expressed only on mRNA level in several cancer cell lines as well as in endothelial cells [1, 30]. The biologic function of TF-A mRNA is unknown [18, 30]. 

Modulation of the differential isoform expression of flTF and asTF as well as TF-A alters the ratio of highly procoagulant TF isoforms (flTF), low- (asTF), and nonthrombogenic forms (TF-A). This—in turn—directly regulates the prothrombogenic potential of vascular cells under (patho)physiologic conditions. The mechanisms of alternative splicing regulation of TF by serine/arginine-rich (SR) proteins or kinases as well as the species-specific differences between human and murine TF were reviewed in detail elsewhere [18, 31, 32]. In this review, we will focus on the functional impact of posttranscriptional regulatory processes, such as alternative splicing. 

Several groups showed modulation of the differential TF isoform expression to alter the prothrombogenic activity of vascular cells. Bogdanov et al. found that regulation of the asTF : flTF ratio on posttranscriptional level influenced the cellular thrombogenicity of human monocytes [33, 34]. In 2006, Schwertz et al. demonstrated that modulation of the alternative splicing apparature altered the expression of prothrombogenic flTF in thrombin-activated thrombocytes (platelets) [35]. We also showed that regulation of alternative splicing processes in human endothelial cells modulated the asTF : flTF ratio as well as the TF-A expression in these cells [1]. This was associated with alterations of endothelial thrombogenicity under proinflammatory conditions [1].

Recently several groups found that miRNAs were also involved in regulation of TF expression and activity [16, 36–38]. In 2011, Teruel et al. suggested that members of the miRNA cluster miR-17~92, such as miR-20a, may be potential modulators of tissue factor expression in cancer cells [38]. In the same year, Zhang et al. showed overexpression of miRNA (miR)-19 to downregulate the tissue factor expression in breast cancer cells [36]. The specific impact of miR-19a and/or miR-19b as well as the impact on the TF isoforms was not analysed in this study. Recently, we demonstrated that selective inhibition miR-19a as well as miR-126 directly induced the expression of both TF isoforms as well as the flTF-mediated cellular thrombogenicity of human endothelial cells [16]. These data suggest miRNAs to modulate the protein expression of TF in different cell types [16, 36]. Moreover, miRNA-mediated posttranscriptional control of flTF expression directly influences endothelial prothrombogenic potential *in vitro *[16]. Further studies, especially those using *in vivo* models, could lead to better understanding of miRNA-mediated control of TF expression and the regulation of TF-associated biologic functions under (patho)physiologic conditions. Moreover, such studies offer the chance to enter a novel field for the possible development of therapeutic starting points and strategies for the treatment of cardiovascular diseases. 

#### 3.1.2. TFPI

Tissue factor pathway inhibitor (TFPI) is another important factor modulator of blood coagulation and thrombogenesis [33, 39]. Human TFPI is expressed in three isoforms (TFPI*α*, *β*, and *δ*) [40]. Alternative splicing leads to modification of the C-terminal structures of TFPI isoforms and differences in their intracellular localization and activity [39, 40]. TFPI*α* represents the soluble “full-length” isoform of TFPI [35]. Alternative splicing leads to the generation of a binding site for glycosyl phosphatidylinositol (GPI) membrane anchor in TFPI*β* [40]. Therefore, this isoform is bound to the membrane of endothelial cells [41]. Recently, Maroney et al. demonstrated that TFPI*β* is a more potent inhibitor of TF-mediated prothrombogenic as well as promigratory activity than soluble TFPI*α* [39]. Due to alternative splicing, a third isoform, TFPI*δ*, is generated from the primary transcript [40]. This isoform contains a new C-terminus but the function TFPI*δ* is unknown [40]. The TFPI*γ* protein isoform was only detected in mice but not in human. In summary, these data indicate that modification of the human TFPI isoform expression under (patho)physiologic conditions can differentially alter the TF-mediated thrombogenicity, due to the different anticoagulant potential of TFPI*α* compared to TFPI*β* [39]. 

Little is known about the impact of miRNA on TFPI expression and function. In experimental cancer settings, miR-616 was found to directly inhibit the expression of TFPI [42]. This was associated with increased tumor growth [42]. Moreover, overexpression of TFPI*α* or TFPI*β*, respectively, led to an altered expression pattern of several miRNAs in breast cancer cells [43]. Therefore, posttranscriptional regulation of TFPI-mediated processes via miRNAs is possible. However, the concrete impact as well as the (patho)physiologic consequences of miRNA-mediated regulatory mechanisms has to be analysed in further studies *in vitro* and *in vivo *in order to estimate the possible therapeutic value.

#### 3.1.3. Other Factors Involved in Blood Coagulation and Thrombosis

Several other factors, such as coagulation factors, are involved in the blood coagulation cascade [44]. Some of these factors, such as FXI and FVIII, were also suggested to be alternatively spliced resulting in the expression of alternative isoforms [33]. 

FXI is involved in the blood coagulation via linking the initiation phase and the amplification phase [44]. FXI circulates in the blood stream [45]. Interaction of FXI with thrombocytes plays an important role under (patho)physiologic conditions, such as in thrombogenesis [45, 46]. Two FXI-like forms were detected in human platelets, whereas only one species was found in plasma [46, 47]. In 1998, Hsu et al. postulated that platelets express an alternatively spliced mRNA species of FXI which could explain the expression of the additional FXI-like form detected in human platelets [48]. In 2004, this hypothesis was challenged by Podmore et al. who used a FXI isoform-specific real-time PCR technique [49]. They found only one FXI mRNA species in thrombocytes and hypothesized that FXI mRNA is not alternatively spliced in platelets [49]. Therefore, it is unclear whether FXI mRNA is alternatively spliced or not. However, recently, Asselta et al. identified several splicing events in the FXI premRNA in human liver and platelets [50]. These splicing events led to nonsense-mediated decay of the generated mRNA species [50]. Moreover, they detected low amounts of one alternatively spliced mRNA variant in human plasma [50]. They concluded that alternative splicing of the primary FXI transcript leads to the expression of a novel FXI isoform [50].

FVIII is also an important factor involved in blood coagulation [49]. This coagulation factor plays an important role under pathophysiologic conditions, such as thrombogenesis and haemophilia A [44, 51]. It was suggested that point mutation-induced alterations of the splicing pattern of FVII premRNA were involved in FVIII deficiency which—in turn—may lead to haemophilia A [51, 52]. In 2010, Shovlin et al. demonstrated that the primary FXIII transcript is alternatively spliced, leading to the expression of four alternative transcripts of FVIII in the endothelium [52]. The authors postulated that the expression of one “full-length” and three catalytically inactive alternatively spliced isoforms could represent a further level of control of the blood coagulation cascade [52]. Moreover, they concluded that endothelial expression of FVIII isoforms is of potential importance to haematological disease states and pulmonary vascular thromboregulation [52].

Fibronectin and its isoforms were also found to affect blood clotting [53]. However, the influence of this factor and its regulation mechanisms are very complex and require a detailed description and interpretation. In this context, we refer to a more detailed review regarding this specific theme [53]. 

The impact of miRNAs on the expression and function of FXI and FVIII is not well studied. Until today, there is only one published paper dealing with the possible association of miRNAs and coagulation factor VIII [54]. The authors described that genetic analyses of haemophilia A patients revealed no association between specific functional mutations in miRNAs and their targets [54]. 

In general, little is known about the role of miRNAs in thrombosis. Most of the literature deals with the impact of miRNAs on platelet biology [55–57]. Expression analyses revealed activation of thrombocytes with thrombin to alter the expression of several miRNAs, such as miR-15 a, miR-495, and miR-98 [55]. In 2009, Landry et al. described, for the first time, that miR-223 directly regulates protein expression of P2Y12 via interacting with the Argonaut 2 protein in human thrombocytes [56]. Recently, another group showed that thrombin-activated platelets were able to deliver functional regulatory Argonaut 2-miR-223 complexes to endothelial cells via microparticles [57]. This—in turn—modulated the expression of miR-223 target mRNAs FBXW7 and EFNA1 in endothelial cells [57].

Together, these data indicate miRNA-mediated regulation processes to be involved in different aspects of prothrombogenic processes under normal as well as under pathophysiologic conditions. But in general, more experimental work has to be done, to get a deeper understanding of these posttranscriptional regulatory mechanism as well as of the (patho)physiologic consequences.

### 3.2. Regulation of the Vascular Tone

Regulation of vascular tone is also an important function of the vasculature under normal as well as under pathophysiologic conditions [58]. Posttranscriptional regulation via alternative splicing and miRNAs was shown to play a significant role for vascular tone modulation (see Tables 1 and 2) [4, 59, 60]. The following part of the paper will describe the influence of posttranscriptional expression regulation on vascular tone control at adequate example molecules, such as eNOS and VEGF. 

#### 3.2.1. eNOS

Nitric oxide (NO) is one of the most important mediators of vascular tone regulation [58]. In the vasculature, NO is synthesized by eNOS [4, 58, 61]. Therefore, eNOS is of immense importance for control of vascular tone under (patho)physiologic conditions [4, 58–61]. Functionally, “full-length” eNOS is composed of a C-terminal reductase domain, which is responsible for electron transfer, and an N-terminal oxygenase domain, responsible for generation of NO from L-arginine. Both domains are linked by a calmoduin-binding domain [58]. Phosphorylation of eNOS as well as formation of eNOS homodimers are key regulators of eNOS activity (NO generation) [58, 61]. Beside transcriptional control, eNOS is regulated on posttranscriptional level [4, 60]. The expression of total eNOS is modulated via miRNAs [60], whereas the isoform expression is regulated by alternative splicing [4, 61].

Constitutive and alternative splicing of the primary eNOS transcript leads to the generation of four isoforms, the “full-length” form of eNOS and the truncated isoforms eNOS13A, B, and C [4, 61]. In contrast to functional “full-length” eNOS, eNOS13A, B, and C possess no reductase domain. Therefore, these isoforms were not able to generate NO [61]. 

In 2007, Lorenz et al. demonstrated that coexpression of truncated eNOS13A with “full-length” eNOS led to the formation of nonfunctional heterodimers (eNOS : eNOS13A) and functional homodimers (eNOS : eNOS) in COS-7 cells [61]. This resulted in the reduction of total eNOS activity dependent on the ratio of inactive eNOS : eNOS13A heterodimers to functional eNOS : eNOS homodimers [61]. We also found that increased expression of eNOS13A, B, and C diminished the NO generation in proinflammatory-induced human endothelial cells [4]. Thereby, regulation of eNOS isoform expression directly affects the generation and bioavailability of NO [4, 58, 61]. This in turn can alter the vascular tone and may also play an important role under pathophysiologic conditions, such as atherosclerosis, endothelial dysfunction, diabetes, and ischemia [4, 58]. Splicing of eNOS can be regulated by different factors. Hui et al. showed that heterogeneous nuclear ribonucleoprotein L modulated alternative splicing of eNOS [62]. In our experiments we found that the serine/arginine-rich (SR) protein kinase DNA topoisomerase I controlled the isoform expression of eNOS13A, B, and C but not the expression of “full length” eNOS in human endothelial cells stimulated with the proinflammatory cytokine tumour necrosis factor (TNF)-*α* [4]. TNF-*α* treatment induced the expression on eNOS13A, B, and C. This was associated with a reduced NO generation in these cells. Inhibition of DNA topoisomerase I abolished the TNF-*α*-induced upregulation of the eNOS isoforms as well as the reduction of eNOS activity [4].

Beside alternative splicing, miRNA-mediated processes were also found to modulate eNOS expression and activity under (patho)physiologic conditions. In 2007, Suárez et al. found that genetic knockdown of the miRNA-processing enzyme Dicer led to an increased expression of eNOS in endothelial cells [63]. This effect was blocked by treatment of cells with miR-221 and miR-222 [63]. In another study, Sun and colleagues demonstrated that miR-155 directly inhibited the eNOS expression on posttranscriptional level [60]. Stimulation of endothelial cells with (TNF)-*α* increased miR-155 expression and downregulated generation of eNOS. This was associated with reduced NO generation and impairment of vascular relaxation [60]. Selective inhibition of miR-155 completely abolished the TNF-*α*-induced blocking of eNOS expression and activity [60]. Recently, another group showed increased expression of miR-21 to decrease expression of PTEN in endothelial cells [64]. This—in turn—led to an elevated phosphorylation of eNOS and increased synthesis of NO [64]. 

These data clearly indicate posttranscriptional modulation of eNOS expression and activity by alternative splicing or miRNAs, respectively, to be of great importance for the regulation of vascular tone.

#### 3.2.2. Other Factors

Several other factors are also involved in modulation of the vascular tone, such as vascular endothelial growth factor (VEGF), myosin light chain (MLC) phosphatase, and guanylyl cyclise-A [58, 65, 66]. Posttranscriptional mechanisms modulate the expression of these factors as well as their function in vascular tone control [59, 66–69].

Alternative splicing of VEGF premRNA leads to the generation of two VEGF isoform families, the VEGF_xxx_a family and the VEGF_xxx_b group [8]. The term “xxx” indicates the number of amino acid in the corresponding protein isoform [8]. The most prominent isoforms are VEGF_165_a and VEGF_165_b. Alternative splicing was found to modulate VEGF-mediated vasodilatation under (patho)physiologic conditions [70, 71]. In 2009, Bills et al. demonstrated that VEGF_165_b blocks the VEGF_165_a-mediated vasodilatation in preeclampsia [70]. This disease is related to pregnancy and characterized by hypertension and endothelial dysfunction [71]. In line with this, Bates et al. also found that VEGF_165_b inhibits vasodilatation mediated via the VEGF_165_a isoform in experimental cancer settings [71]. VEGF is known to modulate the phosphorylation state of eNOS [58]. This—in turn—regulates the activity of eNOS and consequently the release of vascular tone-regulating NO [58, 65]. Therefore, it is possible that modulation of the VEGF isoform expression by alternative splicing may affect phosphorylation and activation of eNOS. Finally, this could modulate the NO-mediated regulation of the vascular tone under (patho)physiologic conditions.

Alternative splicing of other factors was also found to modulate vascular tone control [59, 66]. MLC phosphatase, which contains amongst others the 110–133-kDa myosin-targeting subunit (MYPT1) mediates smooth muscle relaxation in response to NO [59, 72]. Due to alternative splicing, four isoforms were generated which differ in their structure and function [59, 72]. In 2011, Yuen et al. showed regulation of the MYPT1 isoform expression to directly affect the sensitivity of smooth muscle to NO-mediated regulation [59]. Therefore, the authors concluded that modulation of MYPT1 isoform expression determines the response of vascular beds to vasodilators, such as NO, and is crucial for vascular tone regulation under (patho)physiologic conditions [59].

Atrial natriuretic peptides (ANP) are also known to influence blood pressure regulation [66]. ANP binds to guanylyl cyclise-A (GC-A) and induces the generation of cyclic (c)GMP [66]. Hartmann et al. showed that two isoforms of GC-A were expressed in almost all human tissues, a full length of GC-A form and of a GC-A isoform (GC-ADelta) that lacks exon 4 [66]. They found that Angiotensin II induces alternative splicing of GC-A and that the GC-ADelta isoform is not able to bind ANP [66]. Moreover, they demonstrated that GC-ADelta forms heterodimers with wild type GC-A and blocks ANP-induced cGMP synthesis [66]. Therefore, alternative splicing of GC-A premRNA can regulate ANP/GC-A/GMP-mediated signaling and blood pressure modulation [66]. 

Beside alternative splicing, miRNA can also affect regulation of vascular tone on posttranscriptional level [68, 69]. Recently, Somanna et al. showed that the miRNAs miR-1595, miR-2931, and miR-2187 target GC-A expression [69]. Transfection of human embryonic kidney cells with these miRNAs reduced bioavailability of GC-A, ANP-binding to GC-A, guanylyl activity, and accumulation of cGMP in these cells [69]. Thereby, the miRNAs can potentially modulate ANP/GC-A/GMP-mediated signaling and blood pressure regulation under certain (patho)physiologic conditions.

The VEGF pathway is also known to be regulated by miRNAs on posttranscriptional level directly or indirectly. On the one hand, some miRNAs, such as miR-205 and miR-492, were shown to directly inhibit VEGF expression in human endothelial cells and cancer cells [68, 73]. On the other hand, VEGF signaling can be modulated indirectly via miRNA-mediated repression of the expression of VEGF receptor 1 (VEGFR1, FLT1), nuclear factor kappa B (NF*κ*B), and Spred-1 [74–77]. However, little is known about the role of miRNAs in regulation of VEGF-mediated vascular tone control. Only one paper by Fernandes et al. indicated miR-126 to be involved in modulation of NO release and blood pressure control in rat models of hypertension via repressing regulators of the VEGF pathway [77].

Together, these studies indicate alternative splicing as well as miRNAs to be involved in vascular tone regulation via modulating the expression of blood pressure-influencing factors, such as eNOS, VEGF, or GC-A and their isoforms, on posttranscriptional level.

### 3.3. Angiogenesis

The formation of blood vessels (angiogenesis) is also an important vascular function under (patho)physiologic conditions [8, 18]. VEGF as well as TF are known to be of great importance for regulation of angiogenesis [2, 7, 8]. Both factors as well as their angiogenic potential were regulated—at least in part—on posttranscriptional level (see Tables 1 and 2) [2, 8, 16, 73].

#### 3.3.1. VEGF

VEGF is the most important regulator of angiogenesis and other biologic processes under (patho)physiologic conditions [70, 78, 79]. As mentioned before, alternative splicing of the primary VEGF transcript leads to the generation of two VEGF isoform families, the VEGF_xxx_a family and the VEGF_xxx_b group, which differ in their structure and biologic function [8]. Both families, VEGF_xxx_a and VEGF_xxx_b, were found to be of immense importance under normal conditions as well as for pathophysiologic processes of different diseases, such as cancer, preeclampsia, and systemic sclerosis [71, 80–82]. 

The human VEGF_xxx_a group includes VEGF_121_a, VEGF_145_a, VEGF_148_a, VEGF_165_a, VEGF_183_a, VEGF_189_a, and VEGF_206_a [8, 64]. Several studies showed that members of the VEGF_xxx_a family induced proangiogenic processes [7, 8, 28]. 

The family of human VEGF_xxx_b isoforms is formed by VEGF_121_b, VEGF_145_b, VEGF_165_b, VEGF_183_b, and VEGF_189_b [8]. In contrast to the VEGF_xxx_a group, members of the VEGF_xxx_b family exhibit antiangiogenic properties and play an important regulatory role in health and disease [70, 80, 81]. In 2011, Manetti et al. showed overexpression of VEGF_165_b to inhibit VEGF_165_a-mediated activation of VEGF receptor 2 as well as the formation of primary capillary structures by microvascular endothelial cells [80]. Moreover, the same group found that increased generation of antiangiogenic VEGF_xxx_b isoforms and a reduction of proangiogenic VEGF_xxx_a is associated with systemic sclerosis and plays an important role for defective angiogenic processes in those patients [81]. 

Several factors were shown to regulate the generation of pro- and antiangiogenic isoforms by alternative splicing [8, 82]. Modulation of the VEGF isoform expression by these alternative splicing factors was shown to influence (patho)physiologic-relevant biologic functions [8, 83]. In 2008, Elias and Dias found that low pH conditions induced the expression of proangiogenic VEGF_xxx_a forms but not the expression of antiangiogenic VEGF_xxx_b isoforms in endometrial cancer cells [83]. Within the VEGF_xxx_a family, VEGF_121_a was significantly more efficiently induced than the other isoforms VEGF_145_a, _165_a, and _189_a [83]. Moreover, they showed that specific inhibition of splicing-regulatory SR protein (SRp)40, SRp20, and alternative splicing factor/splicing factor 2 (ASF/SF2) reversed the pH-induced effects on the differential VEGF isoform expression [83]. Nowak et al. also found that SRp40 and ASF/SF2 were involved in alternative splicing of VEGF [8]. Additionally, they showed that the SR protein kinase Cdc2-like kinase (Clk)1 as well as SRp55 affected the VEGF isoform expression in human epithelial cells [8]. They found that Clk1 activation specifically induced the expression of VEGF_xxx_b isoforms in transforming growth factor (TGF)*β*1-stimulated cells [8]. Further, the authors demonstrated that overexpression of SRp40 and ASF/SF2 induced VEGF_xxx_a expression, whereas SRp55 increased the generation of VEGF_xxx_b isoforms [8]. The authors postulated that alternative VEGF splicing was differentially regulated via Clk1-mediated activation of SRp55, SRp40, and SF2/ASF in growth factor-stimulated human epithelial cells [8]. Splicing factors, such as SR protein kinase 1 (SRPK1) or SRp55, were also shown to modulate alternative VEGF splicing under pathophysiologic conditions such as in experimental cancer settings [78]. Thus, regulation of alternative splicing modulates the expression of pro- and antiangiogenic VEGF isoforms. This consequently affects the angiogenic potential of cells and tissues under (patho)physiologic conditions.

In other studies, miRNAs were also shown to regulate VEGF expression and angiogenic activity on posttranscriptional level [68, 73]. In 2012, Yue et al. found that miR-205 directly inhibited the expression of proangiogenic VEGF in glioma cells [68]. Recently, Patella et al. demonstrated that miR-492 also binds to VEGF mRNA and reduces its expression in human endothelial cells [73]. This was associated with decreased angiogenesis [73]. Moreover, VEGF-mediated biologic processes, such as angiogenesis, can also be altered by miRNA-induced inhibition of other factors involved in proangiogenic VEGF signaling, such as VEGFR1, NF*κ*B, and Spred-1 [68–71]. Together, these data suggest miRNAs to participate in regulation of VEGF-mediated biologic processes, such as angiogenesis. 

#### 3.3.2. TF

Beside its important role for initiation of blood coagulation and thrombogenesis, TF was shown to mediate proangiogenic effects under (patho)physiologic conditions [6, 84]. TF isoforms, asTF as well as flTF were found to mediate proangiogenic processes via different signaling pathways [6, 28, 29, 84]. In 2008, Versteeg et al. showed that flTF indirectly mediated angiogenesis via activation of FVIIa/protease-activated receptor (PAR)2-dependent signaling [84]. In contrast to flTF, asTF, was found to mediate proangiogenic processes in a PAR2-independent manner [6, 29]. In 2009, van den Berg et al. showed that asTF induced proangiogenic processes, such as cell migration and adhesion of endothelial cells, via integrin *α*v*β*3 and *α*6*β*1 signaling [6]. Further, they demonstrated that treatment of endothelial cells with recombinant asTF induced proangiogenic tube formation *in vitro* and aortic sprouting *in vivo* [6]. Recently, we showed that overexpression of asTF in human A549 cells increased cell proliferation as well as their proangiogenic potential [28]. This was associated with increased expression and secretion of the proangiogenic factors cysteine-rich 61 (Cyr61) and VEGF [28]. Treatment of human microvascular endothelial cells with the supernatant of asTF-overexpressing cells induced proangiogenic tube formation by these cells [28]. Specific blocking of Cyr61 or VEGF significantly reduced the asTF-induced endothelial tube formation [28]. In other studies, we demonstrated that overexpression of asTF induced cell survival, cell proliferation, and the proangiogenic properties of murine cardiomyocytes [29, 85]. This was accompanied with increased expression of proangiogenic Cyr61 and VEGF [29]. Substantiating these results, we also found asTF to stimulate endothelial tube-formation, migration of monocytes, and cardiomyocytic cell proliferation as well as intracellular p38 and ERK1/2 signaling [29]. These processes are known to be involved in angiogenesis [29]. In line with the results of Van Den Berg et al., these processes were also shown to be independent on PAR-2 signaling [6, 28]. As mentioned before, we found asTF to induce expression and secretion of the proangiogenic factors Cyr61 and VEGF [28, 29]. These proteins interact with integrins such as *α*v*β*3 [86–88]. Thus, it seems possible that asTF mediates its proangiogenic biologic functions via direct asTF/integrin interaction as well as via indirect induction of the proangiogenic factors Cyr61 and VEGF under (patho)physiologic conditions.

As mentioned before, alternative splicing regulates the differential isoforms expression of TF as well as the prothrombogenic activity [1, 34, 35, 89]. Recently, we showed that the alternative splicing regulator Clk1 and Clk4 modulated splicing of TF pre-mRNA in hypoxia-treated human A549 cells [28]. Inhibition of these SR protein kinases blocked the hypoxia-induced generation of asTF and flTF and proangiogenic potential of A549 cells [28]. Moreover, Clk inhibition reduced the proangiogenic tube formation by microvascular endothelial cells treated with supernatant of asTF-overexpressing A549 cells [28]. Thus, regulation of the differential TF isoform expression may be an additional posttranscriptional level for the regulation of angiogenic processes.

Beside alternative splicing, miRNAs can also modulate TF-mediated proangiogenic processes. In 2010, Zhang et al. showed that miR-19 inhibited the expression of TF in breast cancer cells [36]. The authors hypothesized that miR-19-mediated regulation of TF expression could affect tumor-associated angiogenesis [36]. Recently, we showed inhibition of miR-19a as well as of miR-126 to induce expression of asTF and flTF in human microvascular endothelial cells [16]. Since asTF and flTF are known to induce proangiogenic processes [6, 28, 29, 84], miRNA-mediated modulation of TF expression may also affect angiogenesis. However, whether this scenario plays a role under (patho)physiologic-relevant conditions is not known and should be investigated in further studies. 

## 4. Concluding Remarks

Together, these studies and data demonstrate that posttranscriptional expression control by alternative splicing and miRNA-mediated mechanisms can modulate important (patho)physiologic-relevant vascular function (see Tables 1 and 2), such as blood coagulation, thrombosis, control of the vascular tone, and angiogenesis, via modulating expression of factors regulating these function, such as TF, eNOS, or VEGF [2, 8, 61, 90]. A better understanding of posttranscriptional expression regulation of those vascular function-regulating factors offers an immense potential for the development of novel therapeutic strategies against cardiovascular disorders and human diseases in general. However, more detailed mechanistic *in vitro* analyses and studies in physiologic-relevant models have to be done *in vivo* do get deeper insights into the regulatory mechanisms and the (patho)physiologic consequences of posttranscriptional expression control. This is an important task for the future. Therefore, the development of novel pharmacologic and molecular biologic tools as well as the improvement of existing techniques is essential to expedite this process.

## Figures and Tables

**Table 1:** Vascular functions of protein isoforms.

Isoform	Vascular functions	References
flTF	High procoagulant activity	[1, 7, 16, 25, 26, 31]
	Angiogenesis	[78]
asTF	Low procoagulant activity	[2, 7, 26]
	Angiogenesis	[6, 27, 28]
TF-A	Unknown	[1, 29]
“Full-length” eNOS	Regulation of the vascular tone via NO generation	[4, 55]
eNOS13A, B, C	No NO generation potential	[4, 55]
VEGF_xxx_a	Proangiogenic	[8, 27]
	Increase vasodilation	[59, 64, 65]
VEGF_xxx_b	Antiangiogenic	[8, 74, 75]
	Reduce vasodilation	[64, 65]
TFPI*α*	Antithrombotic	[34]
TFPI*β*	Antithrombotic	[34]
TFPI*δ*	Unknown	[35]

**Table 2:** Vascular factors modulated by miRNAs.

Protein	Regulating miRNA	References
TF	miR-19a	[16, 32, 33]
	miR-20a	[38]
	miR-126	[16]
TFPI-2	miR-616	[37]
P2Y12	miR-223	[50]
eNOS	miR-155	[54]
VEGF	miR-205	[62]
	miR-492	[67]
VEGF-R1	miR-10	[68]
